# Proprioceptive and Dual-Task Training: The Key of Stroke Rehabilitation, A Systematic Review

**DOI:** 10.3390/jfmk7030053

**Published:** 2022-07-07

**Authors:** Rita Chiaramonte, Marco Bonfiglio, Pierfrancesco Leonforte, Giovanna Loriana Coltraro, Claudia Savia Guerrera, Michele Vecchio

**Affiliations:** 1 Department of Biomedical and Biotechnological Sciences, Section of Pharmacology, University of Catania, 95123 Catania, Italy; pierfra.leonforte@hotmail.it (P.L.); claguerre@hotmail.it (C.S.G.); 2 Provincial Health Department of Siracusa, 96014 Sicily, Italy; mrc.bnf@hotmail.it; 3 Rehabilitation Unit, AOU Policlinico Vittorio Emanuele, 95123 Catania, Italy; loriana92ct@hotmail.it

**Keywords:** stroke, task performance, rehabilitation, systematic review, proprioception

## Abstract

This systematic review aims to reveal the effectiveness of proprioceptive exercise combined with dual-task training in stroke patients. The research was conducted using PubMed, Cochrane Library, Web of Science, and Scopus databases to evaluate studies of rehabilitation interventions with proprioceptive and dual-task exercises in patients with stroke. The keywords for the search were: “stroke” AND “proprioception” OR “proprioceptive” AND “rehabilitation” OR “training” OR “exercises” AND “dual-task” OR “task-performance” with the following inclusion criteria: comparative studies of rehabilitation interventions with proprioceptive and dual-task exercises in stroke patients. Of the 104,014 studies identified, 23 were included according to the inclusion criteria. Proprioceptive and dual-task exercises stimulate and promote postural balance, gait, and quality of life and reduce the risk of falls in stroke patients compared with traditional rehabilitation programs. In conclusion, this systematic review suggests that proprioceptive exercise combined with dual-task training is needed to improve balance and recover gait. Moreover, it provides a comprehensive overview of the literature on the various proprioceptive treatments with contextual dual-task exercises for imbalance after stroke, providing a guide for choosing a complete rehabilitation protocol that combines these two techniques.

## 1. Introduction

Balance is the ability to maintain the correct positioning of the body despite the external environment and stimulation. Proprioception is the sense of position and motion of one’s own body parts and the force generated during movement, which is eessential in coordinating multiple joints and intersegmental movement. It provides feedforward information for motor planning and delivers feedback information for motor response and adaptation to external perturbations [1,2]. There is an increasing interesting in its role in motor learning and neuroplasticity [2]. Accurate sensory feedback, including proprioception, maintains motor control during balance and weight changes in an upright position [3]. For this reason, the ability to maintain balance while walking or during external disturbances is another important goal of rehabilitation, as it is an essential factor in walking. In the cases of sensory impairment or balance perturbation, visual feedback, the vestibular system and proprioception are compensatory strategies to maintain balance [4].

The locomotor pathways that control gait, balance and proprioception are often compromised in stroke patients. In particular, the amount of executive resources required to walk is reflected in prefrontal cortex activity [5]. Stroke patients presented increased prefrontal cortex activation during walking compared with healthy controls due to the increased attentional demand required to achieve locomotion [6]. Adding an additional task to walking increases prefrontal cortex activity and the need to pay attention [5]. Moreover, greater activation of the bilateral superior frontal gyrus, bilateral inferior temporal gyrus, and left caudate nucleus was found in stroke patients during dual-task relative to single-task conditions [6]. A sufficient walking speed of 0.8 ms^−1^ or greater is required to improve dual-task walking after stroke; in fact, only patients with a good walking capacity improved dual-task walking [7]. In particular, walking during contemporaneous cognitive tasks reduces gait speed early after stroke, and this effect often persists even when gait speed improves [8].

Hence, most activities of daily living (ADL) require a combination of cognitive and motor task performance, and these tasks are especially difficult in people after chronic stroke [9]. For this reason, cerebral stroke is a significant cause of disability. Depending on the stage and severity of the disease and based on the localisation of cerebral impairment, stroke can affect consciousness, sensory perception, language, sphincter incontinence, and cognitive and motor abilities, such as postural balance and gait. Stroke imbalance could be due to sensory deficit or motor impairment and is a major cause of functional limitations [10]. About 18% of stroke patients have somatosensory impairment, influencing the prognosis and the outcome of rehabilitation; in fact, about 78% of them report activity loss [11].

Moreover, daily activities in human life require the concomitant combination of motor tasks and cognitive functions while maintaining postural control, standing up or walking [12]. After stroke, these skills fail, and thus, these dual-task abilities are recoverable with specific rehabilitation training [13,14].

It is important to establish an effective rehabilitation strategy that can improve balance, gait, and autonomy and prevent falls, which are unfortunately frequent during dual-task activities.

The literature shows the positive impact of dual-task exercises in the recovery of stroke patients, but very few articles have combined this strategy with proprioceptive training. Thus, it is known that proprioceptive rehabilitation helps in recovering autonomy in gait and stimulating the ability to walk and that dual-task exercises reduce the risk of falls during ADL, but few articles have specified how to combine the two techniques, proposed a standardised duration of training, or described what types of activity could influence the outcomes. For this reason, further studies are necessary. The issue is due to the variability in the definition of proprioceptive exercises and the types of training defined as proprioceptive. This is a widespread problem that has conditioned the research; thus, it is necessary that the reader be aware of this difficulty of correctly defining proprioception. For example, there is little agreement on what exercises are to be included in proprioceptive training. In a systematic review by Aman et al. [15], proprioceptive training is defined as exercises that stimulate the use of somatosensory signals such as proprioceptive or tactile afferents. The authors [15] included proprioceptive interventions for balance training with single- or multi-joint passive and active movement, somatosensory stimulation, and discrimination training and suggested that combined treatments were the most advantageous. The combination of simultaneous visual stimuli, such as paying attention to other tasks, and proprioceptive feedback during gait training may be effective at improving gait after chronic stroke [16]. Cognitive and motor dual-task exercises influence gait and balance in stroke gait, in particular, improving gait parameters, such as speed, stride length, cadence, the score on the Berg Balance Scale, centre of pressure sway area, 2- and 6- min walking test, 10 m and 400 m walking test, and functional independence measure (FIM) [17].

In clinical practice, one of the aims of rehabilitative therapy in stroke is to improve proprioceptive skills, especially to avoid falls, which often occur during dual-task activities. Thus, identifying the best strategies proposed in the literature to recover balance, coordination, and autonomy in ADL helps physicians in clinical practice. This systematic review aims to define proprioceptive strategies combined with dual-task training for the treatment of imbalance and the improvement of postural balance and gait after stroke. The authors collected literature data about the outcomes and the achievement of rehabilitation goals, the duration, the intensity, and the type of proposed proprioceptive training combined with dual-task exercises in stroke patients. Moreover, the secondary aim is to better guide the physician in outlining a rehabilitation protocol by adopting the best strategies described in the current literature.

## 2. Methods

### 2.1. Search Strategy

The literature review search was performed using PubMed, Cochrane Library, Scopus, and Web of Science databases. The specific terms were “stroke” AND “proprioception” OR “proprioceptive” AND “rehabilitation” OR “training” OR “exercises” AND “dual-task” OR “task-performance”. For the Web of Science database, the following filters were applied: in types of documents: articles; in categories: Neuroscience and Clinical Neurology.

Moreover, the reference lists of included studies were screened for additional eligible studies not retrieved by the search. The review period was from August 2021 to February 2022, and the last update was in June 2022. No publication date restrictions were applied. Thus, the search included articles published between 1963 and 2021, and all articles identified in the search were evaluated.

### 2.2. Selection Criteria and Data Extraction

Studies were included if they met the following criteria: (1) original English language articles on imbalance related to stroke, (2) treated with proprioceptive training and contextual dual-task exercises, and (3) subjects with a confirmed stroke diagnosis. The proprioceptive exercises included in the review presented the following characteristics: (1) maintaining balance on a treadmill, considering the need to stimulate proprioception in maintaining the pace of walking based on the tuning of the mobile platform and adapting the gait to the proposed tasks [13,18,19,20,21,22,23,24]; (2) maintaining standing balance on an unstable balance pad [14] or during overground walking, defined as a whole-task practice involving propulsion forward, backward, or sideways or up and down stairs [25,26,27,28,29,30,31], changing speeds [32], using wearable ankle weights [33] or different resistances during gait (i.e., with an elastic band between the legs) [34], or tilting the body and shifting weight from side to side during virtual reality games [35] or aquatic games [36]; (3) proprioceptive neuromuscular facilitation techniques [37]. Both cognitive and motor dual-task exercises were included.

The exclusion criteria consisted of: (1) animal studies, (2) participants with neurological diseases other than stroke, (3) other rehabilitation techniques, and (4) all of the remaining duplicates. (5) Grey literature and unpublished data were not considered.

Two authors (MV and RC) independently reviewed the articles and extracted those that met the inclusion criteria according to the review protocol. In the event of conflicting opinions, consensus was reached after discussion between the authors. They screened the articles by checking titles and abstracts first and then full texts. A large number of papers that had no focus on proprioception or proprioceptive training were excluded. At the end of this first process, an author again reviewed the full texts to make sure that relevant articles were not omitted from the search and retrieved the most recent studies eligible for inclusion. The review followed the Preferred Reporting Items for Systematic review and Meta-Analyses (PRISMA) guidelines [38] for search procedures, study selection, data collection, and analysis and the Participants, Interventions, Comparison, Outcome, and Study Design criteria (PICOS) [39]. In particular, the PICOS criteria used for our research were the following: the participants were older adults after stroke, the interventions were based on proprioceptive rehabilitation therapy and dual-task training, the comparators were different rehabilitation programs, and the outcomes included clinical assessments and diagnostic scales used to assess the recovery of balance and safe gait. The study designs were randomised controlled trials (RCTs) and retrospective, prospective, and observational studies.

The registration number of this systematic review in the PROSPERO platform is CRD42021276239.

### 2.3. Description of the Studies

**Figure 1:**
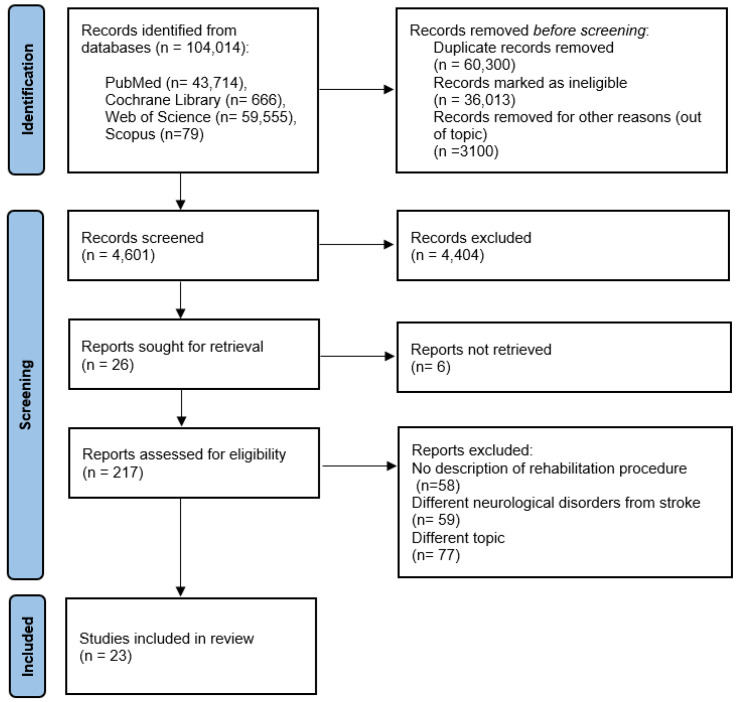
Flowchart of the process of literature search and extraction of studies.

A total of 104,014 articles published from 1968 (the date of the first article found concerning this topic) to 2022 were found using the proposed keywords. After screening titles and abstracts, 217 papers remained for full-text screening, and 23 publications were included in the systematic review. Articles were excluded for the following reasons: 58 did not include the specific rehabilitation procedure, 59 involved individuals with disorders other than stroke, and 77 described different characteristics of stroke or different rehabilitation strategies compared to our topic of research (Figure 1).

The number of studies produced at each stage of the search for the systematic review is shown in Figure 1.

### 2.4. Comparators

The studies described their specific proprioceptive rehabilitation protocol with concomitant dual-task activity and compared it with other exercises: single-task motor exercises, such as gait training [18,26,32,37] or anaerobic exercises with an elastic band [34], a combination of strengthening exercises and gait training [33], bodyweight exercises performed as self-guided rehabilitation at home [25], and balance training with single-task proprioceptive exercises [20,27,28,30,35,40]. Specific rehabilitative protocols such as neurodevelopmental treatment [40] were compared with dual-task proprioceptive training. Cognitive and motor dual-task exercises were compared with each other [13,14]. Motor dual-task exercises with different rehabilitation protocols were compared with each other [36]. Only one article compared the results between motor dual-task exercises and no rehabilitation training [31].

The effectiveness of motor dual-tasks was investigated in most articles, with high variability in dual-task activities (Table 1). Cognitive dual-task training was considered in 5 articles [18,25,34,35]. Cognitive and motor dual-task training were compared in very few articles [13,14]. Cognitive and motor task exercises were conducted separately but in the context of the same rehabilitation program in the same group of patients in 4 articles [14,29,35,37].

Although a quantitative analysis was not possible due to the heterogeneity of the duration and intensity of the training, the measures most frequently used to understand the effectiveness of proprioception and dual-task training were spatio-temporal gait parameters, balance scales, such as the Activities-Specific Balance Confidence Scale (ABC) [41] and the Berg Balance Scale (BBS) [42], and scales that predict the risk of falls and ability/autonomy in walking, such as the timed up and go test (TUG) [43], 10-min walking test (10-MWT) [44], and Functional Ambulation Category (FAC) [45].

### 2.5. Quality of Selected Articles and Outcomes

A final analysis was carried out independently by 2 researchers (MV and RC) to assess the methodological quality of the full texts that met the eligibility criteria. Depending on the type of research study, different tools were used. The papers included in this review are randomised controlled trials (RCTs) and retrospective, prospective, and observational studies, and the scales used were as follows: PEDro (Physiotherapy Evidence Database) [46] and an adapted Sackett’s level of evidence scale [47] from strongest (rating = 1) to weakest (rating = 5), where ranked RCTs are considered the highest level and case series or expert opinions are considered the lowest level (Table S1, Supplementary Materials).

The Grading of Recommendations Assessment, Development and Evaluation (GRADE) guidelines for systematic reviews were used to evaluate the quality of the results [48,49,50,51,52]. The rating of the quality of the study outcome was carried out to indicate the degree of certainty (high, moderate, low, or very low) in the total effect estimates (Table S2, Supplementary Materials).

### 2.6. Risk of Bias

The authors independently assessed the risk of bias in the included studies as low, moderate, unclear, or high risk by considering the characteristics of the Cochrane risk of bias tool [53] and Risk of Bias in Non-randomized Studies of Interventions (ROBINS-I), according to Cochrane methodology [54,55] (Table S2, Supplementary Materials). The points of risk of bias include random sequence generation, allocation concealment, blinding of participants and personnel, blinding of outcome assessment, incomplete outcome data, selective reporting, and other biases.

### 2.7. Statistical Analysis

Due to the heterogeneity and non-uniformity of the data in the included studies, the results are summarised in a descriptive manner.

## 3. Results

### 3.1. Variations in Experimental Conditions across the Studies

A total of 104,014 studies were collected, and 60,300 were excluded due to duplication, 36,013 were ineligible, and 3100 were out of scope. After excluding another 4404 studies because they were not relevant to the topic and including 20 for retrieval, 217 articles were fully assessed for eligibility. Of the 217 selected studies, 194 were excluded because they were not relevant to the study: no description of the rehabilitation procedure (*n* = 58), different neurological disorders from stroke (*n* = 59), and a different topic (*n* = 77).

A total of twenty-three studies were included. These results can be found in the evidence search and selection summary, which is based on the PRISMA flowchart (Figure 1).

None of the study groups were homogeneous for general clinical features, such as clinical presentation, types of gait analysis, balance parameters, and scales examined (Table 1).

There was a large variation among studies in the duration of the disease, the time of the first examination of imbalance, treatment duration, and the follow-up period at the end of therapy.

### 3.2. Study Characteristics

The sample characteristics and design details of each included study are shown in Table 1.

The samples included subjects with chronic stroke, which took place at least 6 months before the proposed rehabilitation. In two articles, the individuals had suffered from stroke 3 months before [20,35].

### 3.3. Summary of Findings

Stroke patients present an increased risk of falls due to diminished proprioception, balance, and dual-tasking ability. For determining the contribution of proprioceptive signals for balance control, many biomechanical measures have been employed, such as latencies and amplitudes of electromyographic signals, joint kinematics or kinetics, or variables indicative of the postural sway of the body’s centre of mass. With respect to proprioceptive training, this means that an intervention focusing on training the proprioceptive sense may train one or both aspects of proprioception, that is, the conscious perceptual aspect or the unconscious or implicit sensorimotor aspect [15]. Thus, proprioceptive and dual-task exercises play an important role in stimulating and promoting postural balance, gait, and quality of life as well as reducing the risk of falls, but the current literature describing these rehabilitation protocols is limited. In fact, research shows excessive variability in the quality, intensity, and duration of training. Furthermore, there are no specific protocols based on the severity of the imbalance. According to our research, to date, the severity of postural imbalance and the estimation of the risk of falling do not modify the therapeutic approaches of physicians.

The most frequently used measures for affirming the efficacy and usefulness of proprioceptive and dual-task training are spatio-temporal gait parameters, balance scales, such as the Activities-Specific Balance Confidence Scale (ABC) and the Berg Balance Scale (BBS), and scales that predict the risk of falls and ability/autonomy in walking, such as the timed up and go test (TUG), 10-min walking test (10-MWT), and Functional Ambulation Category (FAC). Unfortunately, the difference in the duration, intensity, fatigability, and adherence to the treatment made a statistical comparison of the results impossible. Moreover, the level of fatigability and adherence to treatment, which can be compromised, for example, by possible post-stroke depression, were rarely shown.

### 3.4. Proprioceptive Rehabilitation Program and Dual-Task Exercises

The 23 selected articles are described on the basis of the proprioceptive rehabilitation examined in each study. Table 1 shows the proprioceptive strategies combined with dual-task training described in the current literature.

Regarding the kind of proprioceptive exercises, most authors proposed maintaining standing balance on an unstable balance pad [14] or during overground walking (that is, a whole-task practice involving propulsion forward, backward, or sideways or walking up and down stairs) [25,26,27,28,29,30,31], changing speeds [32], using wearable ankle weights [33] or an elastic band between the legs [34], or tilting the body and shifting weight from side to side during virtual reality games [35] or during aquatic games [36]. Other authors described exercises for balance on a treadmill, considering the need to stimulate proprioception in maintaining the pace of walking based on the tuning of the mobile platform and adapting the gait to the proposed tasks [13,18,19,20,21,22,23,24]. The proprioceptive neuromuscular facilitation technique during dual-task training was described too [37].

The proposed dual-task exercises, executed contemporary to proprioceptive training, consisted of (1) cognitive activities, such as auditory [37] or visual cues [34] that triggered the action, performing arithmetic operations [13,14,18,20,22,29], counting backward [24], matching words [25], exercises for verbal fluency [28], memory tasks [21,30,35], exercise imagery [27], and talking about planning activities [23]; (2) motor activities, such as writing [32], moving an object (cups, coins, sandbag, or balls) [13,14,19,26,30,31,33,36,40], avoiding obstacles [20], and playing Wii Fit games [35].

Most articles obtained significant results after training 3 days a week for 4 weeks [25,29,31,32,34,37], and other articles proposed continuing exercises for more than 4 weeks: for 6 [14,18,35,36] or 8 weeks [13]. Other studies presented their results after more sessions of training in a week: 4 days a week for 4 weeks [33], 5 days for 4 weeks [21,22,26], 5 days a week for 6 weeks [40], or 5 days a week for 8 weeks [27]. Other authors obtained the same positive results with fewer exercises, only 5 days of training [30], or with fewer sessions in a week: 2 days a week for 4 weeks [19], 5 weeks [20,24], or 8 weeks [23] and only a day a week for 8 weeks [28].

The duration of each session was very variable, and it was not explained why; the resistance and the clinical conditions of patients probably conditioned the duration of each sitting. Thus, most studies proposed that each session last 30 min [13,14,21,22,23,25,26,27,31,32,34,40], and only one article suggested 15 min for each sitting [37]. Most authors proposed longer sessions of 35 min [19], 40 min [36], 60 min [18,28,29], 90 min [20,24,35], or 110 min [30].

The heterogeneity regarding the types of exercises and the timing did not permit suggesting a protocol of proprioceptive and dual-task exercises, but despite that, this combined training has been shown to be very effective in restoring balance during ADL.

## 4. Discussion

### 4.1. Summary of Collected Data

To our knowledge, there are very few studies in the current literature that report dual-task exercises in the context of proprioceptive rehabilitation strategies in stroke patients. For this reason, our systematic review collected data related to all of the different strategies that combined the two different types of training.

In fact, proprioception and multi-task abilities are essential to ensure autonomy in ADL. After stroke, the impairment of proprioception and dual-task abilities could cause severe disability that is correctable with specific rehabilitation. Though conventional training methods facilitate balance control, dual-task workouts with motor and cognitive exercises together with proprioceptive training could lead to recovery and reduce the risk of falls. Our study, supported by the international literature, suggests the importance of promoting specific protocols and developing guidelines about proprioceptive rehabilitation combined with contextual dual-task strategies.

The analysed samples included mostly subjects with chronic stroke; after the clinical condition has stabilised, the rehabilitation program has the main goal of the recovery of autonomy in ADL and social reintegration. Task-oriented proprioceptive training is proposed as effective rehabilitation to reach this objective.

### 4.2. Task-Oriented Rehabilitation Therapy in the Context of Proprioceptive Rehabilitation

ADL require a combination of cognitive task performance and motor task performance, especially during postural balance and ambulation [31]. Therefore, balance and gait in stroke patients reflect changes in the motor and cognitive abilities required in the dual activities of daily life. Moreover, there is a significant correlation between proprioception impairment, difficulty in dual-task activities, and increased risk of falls after stroke [56]. In fact, the deterioration of balance control causes falls in post-stroke individuals, particularly during dual-tasking, suggesting that improving task performance is the goal for rehabilitation in proprioception impairment [30]. Two studies [25,37] highlighted that during daily dual-task cognitive performance, such as decision making, visuospatial memory, and working memory, the risk of falls while walking increased. For this reason, specific training improves daily dual-task activities and, consequently, autonomy in ADL.

Several rehabilitative strategies have been studied to prevent falls and improve balance, gait performance, and participation in the community. In fact, in the current literature, there is an increased interest towards the effect of dual-activity training in older adults [57] and in patients with dementia [58], Parkinson’s disease [59], and multiple sclerosis [60], as well as those with stroke, as documented by the articles in this systematic review. Motor and cognitive multi-task exercises that focus on the principles of motor learning and plasticity and have the aim of transferring the gains from the clinic to daily life have an important place in stroke rehabilitation [61]. Task-oriented exercises that provide the opportunity to participate in real-world events and that include tasks requiring both cognitive and motor tasks support adaptation and participation in life situations in chronic stroke patients compared to only motor problem-focused training [61]. Thus, dual-task training promotes autonomy in ADL by improving the ability to process information. Despite this, only a few reports, shown in Table 1, studied which dual-task exercises could be most effective in the context of proprioceptive rehabilitation for improving balance and daily skills after stroke. According to our research, the high heterogeneity of the proposed exercises did not influence the outcomes; in fact, all of the proposed cognitive and motor task exercises combined with proprioceptive exercises, such as maintaining balance on a mobile platform [13,18,19,20,21,22,23] or unstable balance pad [14] or during overground walking [25,26,27,28,29,30,31], changing speeds [32], wearing ankle weights [33], or using elastic band between the legs [34], were effective. The variability in the organisation of the sessions of rehabilitation, especially regarding the timing, highlighted the positive responses of patients to treatments for an average of 3 days a week for 4 weeks [25,29,31,32,34,37], although a shorter or longer period did not modify the results.

### 4.3. Be on Guard

A few studies [19,21,30,35] proposed the combination of proprioceptive and dual-task training with a virtual reality setting.

Beyond the classic methods, i.e., the proprioceptive neuromuscular facilitation technique [37], new technologies are stimulating new interest, such as rehabilitation programs using virtual reality games. Subramaniam et al. [30] and Kannan et al. [35] used a program based on virtual reality as a form of dual-task training. Training with virtual reality exercises presented additional cognitive tasks, such as semantic memory or divided attention activities. Furthermore, it is shown that virtual reality increases motivational levels and improves physical function, volitional control of stability, and semantic and working memory [30].

Although virtual reality and robot-assisted gait systems are widely preferred technology-supported rehabilitation methods in stroke, no studies with these new technologies have been found yet. It is necessary to focus on more than one task while walking in daily life, and this becomes a difficult task to cope with for stroke patients. As in virtual reality games, adding an additional task to gait training is an example of the multi-task feature of walking activity in everyday life and leads to the distraction of the patient [61]. VR training in stroke patients increased movement quality and functional capacity [62], while robot-assisted gait training improved gait and balance [63]. The combined therapy motivates patients and increases active participation in the rehabilitation of stroke patients [64].

In sum, the rehabilitation of proprioception combined with simultaneous dual-task training, including in virtual reality settings, definitely improves balance and postural control, walking, and gait speed and prevents falls.

### 4.4. Enjoy Dual-Task and Proprioceptive Training

Enjoyment of the rehabilitation program could improve results. Two studies in which rehabilitation had an important motivational component were in the context of aquatic games [36] and tango lessons [65]. These studies allowed patients to enjoy their rehabilitation program and improved treatment adherence and final outcomes. The tango training reported by Hackney et al. [65] was not included in the systematic review because it is a case report, but it is described in the text. Indeed, dancing, in particular tango, adapted to a dual-task proprioceptive program for a subject with stroke, could encourage the subject to not give up the program prematurely and achieve the set goals [65].

## 5. Limits

The heterogeneity related to the study design did not allow us to obtain quantitative results, such as the different outcomes measured across the small number of existing reports. The lack of information about some clinical characteristics, such as comorbidity, post-stroke depression, level of fatigue, and the consequent physical adherence of subjects, limited the possibility of obtaining more complete outcomes, which might be a confounding factor and could affect results. Moreover, the articles that included dual-task training used different cognitive and motor tasks, making the sensitivity of different modalities unclear.

## 6. Conclusions

It is known that the impairment of proprioception after stroke is strongly associated with falls. In addition, dual-task activities increase the risk of falling among stroke subjects. This systematic review provides a comprehensive overview of the literature on all possible different dual-task training types in combination with proprioception intervention in subjects after stroke. According to this search, despite the heterogeneity of the treatments, i.e., the duration, the kinds of exercises, and the scales and scores used to analyse the results, all of the articles included in this review obtained significant results regarding balance and reduction in falls in ADL after proprioceptive and dual-task exercises.

Personalised dual-task training in combination with proprioceptive intervention is important for improving motor control and motor learning by automating gait. Other studies are needed in this area to compare different rehabilitation strategies and find the best protocol for this disorder.

## Figures and Tables

**Table 1:** Rehabilitation programs after stroke for postural imbalance, proprioception, and dual-task training. Characteristics and outcomes of studies included in the systematic review, according to PICOS criteria for inclusion of studies.

Authors, yr	Study Design	Sample Size, y	Months after Stroke	Outcomes Measure	Rehabilitative Therapy	Duration Therapy	Exercise Description	Results after Therapy
Ada 2003 [25]	CLT	A: 13 p B: 14 p 66 ± 11 y	>6	Walking speed, distance, step length, step width, cadence, SA-SIP30	A: Cognitive task and walking program B: Home exercises	3 d/w, for 4 w	Cognitive task on treadmill and overground walking program for 30 min. Proprioceptive group walked around an outdoor circuit, such as curbs, slopes, stairs, and rough terrain, while performing a cognitive dual-task. The cognitive task consisted of matching the word “red” with the response “yes” or the word “blue” with the response “no”. For the control group: home exercise program, such as stretching and strengthening exercises of lower limbs and training for balance and coordination.	Effectiveness of treadmill and overground walking training. Gain of 18 cm/s in stroke patients
An 2014 [13]	CLT	A: 12 p B: 12 p C: 12 p	Chronic stroke	STI, WDI with open and closed eyes, TUG, FSST, 10 MWT	A: Motor DT gait training B: Cognitive DT gait training C: Motor and cognition DT gait training	3 d/w, for 8 w	Fifteen minutes of walking on the treadmill, with motor or cognitive tasks or both types of tasks (30′). The motor tasks included: “tossing up and catching a ball”, “rehanging loops on different hooks”, “doing up buttons after unbuttoning”, “holding a cup of water without spilling it”, and “receiving and returning a cup of water”. Dual cognitive tasks included: “discerning colours”, “mathematical subtraction”, “verbal analogical reasoning”, “spelling words backward” and “counting backward”.	Motor and cognitive DT training improved motor performance and balance and gait abilities
Baek 2021 [18]	CLT	A:17 p B: 17 p	Chronic stroke	Gait parameters (speed, stride, variability, and cadence), CRR, DTC, FES	A: Gait training on a treadmill and cognitive task exercises at the same time B: Separately, first gait training on a treadmill, then cognitive task exercises	2 d/w, For 6 w	Sixty minutes of treadmill training with a cognitive task: serial subtraction by three from two-digit numbers randomly selected	Dual-task gait treadmill training was more effective in improving gait ability in dual-task training and dual-task interference than single-task training involving gait and cognitive tasks separately in subjects with chronic stroke
Cho 2015 [21]	CLT	A: 11 p 60.0 y B: 11 p 58.6 y	Chronic stroke patients A: 273.9 d B: 263.9 d	Spatio-temporal gait parameters: speed, cadence, step and stride length	A: Virtual reality training with cognitive task B: Virtual reality treadmill training	5 d/w, for 4 w	The standard rehabilitation program consisted of physical and occupational therapies. Thirty minutes/session of treadmill and cognitive tasks (memory, arithmetic, and verbal tasks)	Effectiveness of virtual reality training with cognitive load L on walking function under the dual-task condition
Choi 2014 [37]	CLT	A: 19 p 49.1 11.9 B: 18 p 49.2 ± 7.05 y	18.2 ± 5.7	TUG, sway velocity	A: Cognitive–motor dual-task B: Single-task training	3 d/w, for 4 w	The dual cognitive–motor task group performed rehabilitation for 15 min with a random auditory cue while walking on a treadmill. The sound of a bell was the auditory cue indicating that the participants should move the circle ring from side to side. The single-task group walked on the treadmill. Conventional physical therapy included progressive resistance exercise and postural control, neurodevelopment and occupational training, Brunnstrom movements, and proprioceptive neuromuscular facilitation techniques.	Cognitive and motor dual-task training and auditory cues improved balance
Fishbein 2019 [19]	CLT	A: 11 p B: 11 p 65.2 ± 9 y	>12	10 MWT, TUG, FRT LRT-L/R, ABC, BBS	A: DT training B: Single-task treadmill walking	2 d/w, for 4 w	Mobilisation and flexibility exercises for 8 min and walking around for 3 min, treadmill for 3 min. Subsequently, the single-task group walked for 20 min, and the dual-task training group, while walking for 20 min, trained with 3 virtual games: hit the virtual ball, touch the virtual boxes, clean the virtual windows	Improvements in BBS, FRT, LRT-L/R, 10 MWT, ABC in A group; in gait variables. No changes in TUG.
Her 2011 [14]	RS	A: 12 p B: 13 p C: 13 p 64.8 ± 5.2 y	>12	BBS, FIM, centre of pressure	A: MDT B: CDT C: MCDT	3 d/w, for 6 w	Group A: Exercises comprising motor tasks for 30 min, such as exchanging a ball, receiving a ball with a basket, bouncing a ball on the floor, and holding a glass with water and exchanging a water glass while maintaining balance on an unstable balance pad. Group B: Exercises of cognitive tasks such as counting backwards, calculating two subtractions, calling the correct names of objects, and reciting words in reverse order while maintaining balance. Group C: Motor and cognitive exercises with postural control task.	To obtain improvement in balance and gait, both motor and cognitive DT were needed, not only motor or cognitive DT alone.
Hong 2020 [34]	CLT	A: 8 p 56.6 ± 8.7 y B: 9 p 66.2 ± 11.5 y	>6	TUG, BBS	A: CDT B: General task group	3 d/w for 4 w	Exercises with a cognitive task for 30 min. The program consisted of maintaining a standing posture while moving the lower extremity of the less-affected side toward the 3 flexion directions of the hip joint and then moving it back in place.	Cognitive task training is a more effective intervention method to improve balance and gait ability after stroke.
Iqbal 2020 [33]	CLT	A: 32 p 58.2 ± 7.13 y B: 32 p 58.8 ± 6.13 y	Chronic stroke	Step length, stride length, 10 MWT, TUG, cycle time, cadence	A: Motor dual-task training B: Conventional training	4 d/w for 4 w	Motor dual-task with exercises were conducted for 40 min. Slowly walking backward, sideways, and forward on a smooth surface while holding a 100 gm sandbag.	Conventional physical therapy and dual-task training effectively improved the gait ability of chronic stroke patients, who showed a significant improvement in all spatial and temporal gait variables.
Kannan 2019 [35]	CLT	A: 13 p B: 12 p 59.2 ± 6.3 y	>3	TUG, 6 MWT, ABC, BBS	A: High-intensity, tapered motor and cognitive rehabilitation B: Conventional balance rehabilitation	10 sessions, for 6 w	The cognitive tasks and proprioceptive exercises included four Wii Fit games played for 5 min. The control group performed balance training exercises for 90 min: 10 min of stretching, 15 min of functional stretching, 35 min of balance training, and 10–15 min of treadmill walking. The cognitive tasks included: training for semantic memory (i.e., recite as many types of animals as they could within the time limit provided), verbal fluency (i.e., participants recited words that began with the letter provided, such as “A”, while avoiding saying proper nouns), abstract memory (i.e., participants completed phrases according to the relationship of the cue in the sentence), and repetition of letters.	Effectiveness of cognitive and motor rehabilitation in improving balance control and cognition
Kim 2013 [26]	RS	A:14 p B: 15 p 56.4 ± 12.3 y	7 ± 2.4 m	Cadence, speed, step time, cycle time, step length, stride length	A: Conservative physical therapy B: Dual-motor task training	5 d/w, for 4 w	Neurological developmental treatment for 30 min. The motor dual-task training included passive and active resistance exercises and exercises for coordination, motor sensation, and balance. The proprioceptive neuromuscular facilitation lower limb patterns consisted of: flexion–adduction–external rotation knee flexion or extension–adduction–external rotation knee extension. Dual-task: rising from a chair from the sitting position while picking up plastic cups that lay in front of the feet, then slowly walking forward, sideways, and backward on a flat surface while holding a 100 g sandbag against the affected wrist and going up and down a ramp or stairs while transferring cups from tables of different heights located beside the ramp or stairs in consecutive order.	Improvement in temporal (cadence, speed, step time, and cycle time) and spatial parameters (step length and stride length) in a DT group
Kim 2014 [32]	RS	A: 10 p B: 10 p 68.5 ± 7.8 y	17.9 ± 13	Stroop test, TUG, 10 MWT, F8WT	A: Traditional rehabilitation program B: Traditional rehabilitation program + DT training	A: 5 d/w 1 w B: 3 d/w, 4 w	Motor dual-task training was conducted for 30 min and consisted of words written using inks of various colours, with the subjects having to state the colour of the ink and gait tasks, which included walking on a level surface, walking while changing gait speeds, walking with vertical or horizontal head turns, walking with pivot turns, stepping over or around obstacles, and walking up and downstairs.	Improvement in cognitive skills and gait after DT training
Kim 2016 [40]	RS	A: 10 p B: 10 p 68.5 ± 3.1 y	10.9 ± 1.1	BBS, 5-Times Sit-to-Stand Test, Functional Reach Test, TUG, 10 MWT, FGA	A: Neurodevelopmental treatment + aquatic dual-task training B: Neurodevelopmental treatment	5 d/w, for 6 w	Aquatic motor dual-task training for 30 min a day, including stability exercises, such as standing with eyes closed, stability exercise while playing catch with the therapist, walking 10 m at a comfortable speed, and walking 10 m at a comfortable speed while holding a 200 mL cup of water	Improvement in balance and gait after aquatic DT training
Kim 2018 [22]	CLT	A: 13 p 52.62 ± 9.84 y B: 13 p 56.15 ± 10.82 y	Chronic stroke	Speed, cadence, single support time, stride length, 10-MWT	A: Progressive treadmill cognitive dual-task gait training B: Conventional treadmill gait training	5 d/w, for 4 w	Thirty minutes of treadmill exercises with cognitive dual-task: speaking numbers task, arithmetic subtraction, memory task, and verbal fluency	Progressive treadmill cognitive dual-task gait training had a positive influence on the gait and clinical gait.
Lee 2015 [27]	RS	A: 18 p B: 18 p 28 p < 65 y 8 p > 65 y	11.5 ± 1.9	BBS, TUG, joint position sense	A: Motor imagery exercises for 5′ and proprioceptive rehabilitation for 25′ B: Proprioceptive rehabilitation for 30′	5 d/w, for 8 w	Motor image training and proprioceptive training for 30 min included exercises on a balance pad with 5 different tasks. The program included: standing with the support position of two feet and standing upright by moving both heels up and down. Stretching and eyes-closed exercises, balance board exercises shifting weight left and right and forward and backward to the maximum, and sitting and standing on a balance board	Improvements in K-BBS, TUG, weight-bearing ratio, and joint position sense error in group A > B
Meester 2019 [23]	CLT	A: 26 p 60.85 ± 14.86 y B: 24 p 62.25 ± 15.53 y	>6 months	SF-36, EuroQol-5D-5L, PASE, step activity.	A: Traditional treadmill B: Treadmill with dual-task	2 d/w, for 10 w	Thirty-minute treadmill program at an aerobic training intensity with dual-task: listening task or talking about planning daily activities	Walking with specific additional cognitive distraction (dual-task training) might increase activity more over 12 weeks.
Pang 2018 [28]	CLT	A: 25 p B: 28 p 61.2 ± 6.4 y	75.3 ± 64.9	ABC, Frenchay Activities Index, Stroke-specific Quality of Life Scale	A: DT balance/mobility training B: Single-task balance/mobility training	1 d/w, for 8 w	For 60 min a week, the motor dual-task program included walking combined with verbal fluency and with serial-3-subtractions and the timed up and go test combined with verbal fluency.	After DT rehabilitation, improvement in speed, reduction in falls, and no improvement in social participation and quality of life
Plummer 2021 [29]	CLT	A: 18 p B: 18 p	<3 years	Gait speed, cognitive task performance	A: Dual-task gait training B: Single-task gait training on cognitive–motor dual-task	3 d/w for 4 w	Cognitive task performance during walking included spontaneous speech, arithmetic word, backward spelling, working memory, random number, calculating time, backward numbers, and naming opposites. The exercises lasted 60 min.	Both single- and dual-task gait training improved single- and dual-task gait speed but did not change the amount of relative interference.
Saleh 2019 [36]	CLT	A: 25 p B: 25 p 49.7 ± 1.8 y	9.02 ± 1.8	Stability index, speed, step length, time of support on limb	A: Motor DT in water B: Motor DT on land	3 d/w, for 6 w	The dual-motor task training included exercises performed during walking: holding a ball, holding a 200 mL cup of water and standing on a balance board, walking forward, walking sideways and walking backward in each condition, and transferring coins from one pocket to another. The exercises lasted 45 min.	Improvement in anteroposterior and mediolateral stability index, speed, step length, and time of support on the affected limb after water exercises
Subramaniam 2014 [30]	RS	8 p 51.75 y	6.1 ± 4	BBS, TUG, IMI Reaction time, speed, maximum excursion, directional control	Virtual reality balance training in DT	110′/d, for 5 d consecutive	Cognitive motor dual-task training for balance in virtual reality consisted of 110 min of balance board games: table tilt, tightrope, soccer, and balance bubble, played while performing cognitive tasks, such as memory tasks, word list generation, letter–number sequencing, and question–answer and memory recall games.	Improvement in balance after DT rehabilitation
Timmermans 2016 [20]	CLT	A: 20 p B: 20 p	>3 m	10 MWT, TUG, FAC, BBS, ABC	A: Treadmill-based C-Mill therapy B: Overground Falls program	2 d/w, for 5 w	A group performed cognitive–motor dual-task training: the C-Mill treadmill training program, which consisted of 1.5 h each session, twice a w for 5 w. It included practising avoidance of visual obstacles, practising accurate positioning of the foot on a step-to-step basis, walking forwards in a regular or irregular sequence of visual stepping objectives, exercises to practise acceleration and deceleration while maintaining position, and playing a functional and interactive adaptability walking game. The FALLS program consisted of an overground therapy program to reduce the number of falls by practising walking adaptability. It included exercises to practise obstacle avoidance, exercises to practise foot placement while walking over uneven terrain, and tandem walking and slalom. These exercises were also performed under visual constraints.	Improvement after C-Mill therapy with respect to FALLS program
Timmermans 2021 [24]	CLT	A: 16 p 52 ± 13 y B: 17 p 59 ± 10 y	>3 months	Speed, 10 MWT	A: Treadmill-based C-Mill therapy B: Overground Falls program	2 d/w, for 5 w	Ninety minutes/session. A group performed cognitive–motor dual-task training: the C-Mill treadmill training program. The FALLS program for the other group consisted of exercises for walking adaptability.	Greater improvement in context-specific walking speed in C-Mill group
Yang 2007 [31]	CLT	A: 12 p B: 13 p 59.3 ± 11.8 y	64.5 ± 63.1	Speed, cadence, stride time, stride length, temporal symmetry index	A: Ball exercise program B: No rehabilitation training	3 d/w for 4 w	Motor dual-task program: 30 min of gait training while manipulating 1 or 2 balls with diameters of 45, 55, 85, and 95 cm and a basketball. The training program included walking while holding 1 or 2 balls in both hands, walking to adapt to the rhythm of bouncing 1 ball with 1 hand or both hands, walking while holding 1 ball in 1 hand and simultaneously kicking another ball from basketball into a net, and walking while bouncing a ball with both hands. Three motor tasks of simple walking included walking with buttoning task and walking with the task of carrying a cup on a tray.	Walking ability was significantly improved after training. Gain of ba29.74 cm/s.

Patients post stroke: p; group A: A; group B: B; group C: C; years old: y; female: f; male: m; years: y; days: d; hours: h; months: m; minutes: min; weeks: w; clinical trial: CLT; observational study: OS; dual-task: DT; task-specific motor relearning program: MRP; electromyography: EMG; retrospective study: RT; Motor Assessment Scale: MAS; the Sødring Motor Evaluation Scale: SMES; Nottingham Health Profile: NHP; stability test index: STI; weight distribution index: WDI; timed up and go test: TUG; four square step test: FSST; 10 m walk test: 10MWT; 6 min walking test: 6 MWT; Berg Balance Scale: BBS; Activity-specific Balance Confidence: ABC; Functional Ambulation Category: FAC; Functional Reach Test: FRT; Lateral Reach Test Left/Right: LRT-L/R; One-Leg Stand Test: OLST; Intrinsic Motivation Inventory: IMI; Functional Gait Assessment: FGA; Figure-of-8 Walk Test: F8WT; Subjective Index of Physical and Social Outcome: SIPSO; stroke-adapted 30-item version of the Sickness Impact Profile: SA-SIP30; Medical Research Council scale: MRC; Fugl-Meyer Upper Extremity scale: F-M UE; Functional Independence Measure scale: FIM; correct response rate: CRR; dual-task cost: DTC; Fall Efficacy Scale: FES; Rivermead Mobility Index: RMI; Range of Motion: ROM; Physical Activity Scale for Elderly: PASE.

## Data Availability

The data presented in this study are available on request from the corresponding author.

## Supplementary Materials

The following are available online at https://www.mdpi.com/article/10.3390/jfmk7030053/s1.
