# Supplementary material for: Proprioceptive and Dual-Task Training: The Key of Stroke Rehabilitation, A Systematic Review
Source: J Funct Morphol Kinesiol. 2022 Jul 7;7(3):53. doi: 10.3390/jfmk7030053 (PMC9326539; doi:10.3390/jfmk7030053)
Supplement: Supplementary file 1 [file jfmk-07-00053-s001.zip › jfmk-1775669-supplementary.pdf]

Journal of
Functional Morphology and Kinesiology

MDPI

Table S1. Evidence level based on adapted Sackett's levels of evidence.

Level	Study type	Studies
1a	RCTs showing similar findings (PEDro Scale score ≥6)	Ada 2003 [25], An 2014 [13], Baek 2021 [18], Cho 2015 [21], Choi 2014 [37], Fishbein 2019 [19], Hong 2020 [34], Iqbal 2020 [33], Kannan 2019 [35], Kim 2018 [22], Meester 2019 [23], Pang 2018 [28], Plummer 2021 [29], Saleh 2019 [36], Timmermans 2016 [20], Timmermans 2021 [24], Yang 2007 [31]
1b	RCTs with Narrow Confidence Intervals 3 (PEDro Scale score ≥6)	-
2a	RCT (PEDro Scale score =4-5)	-
2b	non-randomized controlled trial, Cohort Studies, Retrospective studues	Her 2011 [14], Kim 2013 [26], Kim 2014 [32], Kim 2016 [40], Lee 2015 [27], Subramaniam 2014 [30]
2c	Outcomes Research	-
3a	Case-Controlled Studies	-
3b	3B Case-controlled Study	-
4	Case Series, Poor Cohort Case-Controlled, Conflicting evidence of two or more equally designed studies	-
5	Expert Opinion, RCTs with PEDro scores ≤ 3, case studies/case descriptions	-

Controlled Trials RCTs.

Table S2. Risk of bias summary for each included study and GRADE quality of evidence.

Studies, year	Random sequence generation	Allocation concealment	Blinding participants	Blinding of outcome assessment	Incomplete data	Selective reporting	Other bias	Risk of bias
Ada 2003 [25]	+	+	+	+	+	+	+	Low
An 2014 [13]	+	+	-	-	+	+	+	Low
Baek 2021 [18]	+	+	-	-	+	+	+	Low
Cho 2015 [21]	+	+	-	-	+	+	+	Low
Choi 2014 [37]	+	+	+	+	+	+	+	Low
Fishbein 2019 [19]	+	+	+	+	+	+	+	Low
Hong 2020 [34]	+	+	-	-	+	+	+	Low
Iqbal 2020 [33]	+	+	-	-	+	+	+	Low
Kannan 2019 [35]	+	+	-	-	+	+	+	Low
Kim 2018 [22]	+	+	-	-	+	+	+	Low
Meester 2019 [23]	+	+	-	-	+	+	+	Low
Pang 2018 [28]	+	+	+	+	+	+	+	Low
Plummer 2021 [29]	+	+	-	-	+	+	+	Low
Saleh 2019 [36]	+	+	-	-	+	+	+	Low
Timmermans 2016 [20]	+	+	+	+	+	+	+	Low
Timmermans 2021 [24]	+	+	-	-	+	+	+	Low
Yang 2007 [31]	+	+	+	+	+	+	+	Low

Studies, year	Bias due to confounding	Bias in selection of participants for the study	Bias in classification of interventions	Bias due to deviations from intended intervention	Bias due to missing data	Bias in measurement of outcomes	Bias in selection of the reported result	Risk of bias
Her 2011 [14]	+	+	+	+	+	+	+	Low
Kim 2013 [26]	+	+	+	+	+	+	+	Low
Kim 2014 [32]	+	+	+	+	+	+	+	Low
Kim 2016 [40]	+	+	+	+	+	+	+	Low
Lee 2015 [27]	+	+	+	+	+	+	+	Low

Subramaniam 2014 [30]	+	+	+	+	+	+	+	Low

Quality assesssment					Summery of findings	Quality of Evidence GRADE
N. of studies	Limitations	Inconsistency	Indirectness	Pubblication bias	Sample characteristics	
23 studies	No significant limitations	No serious inconsistency	No serious indirectness	Unlikely	Population: Stroke adults<br>Intervention: Proprioceptive dual-task training<br>Comparison: Rehabilitation strategies<br>Outcomes: Improvement of balance, gait, autonomy	High

+ indicates reporting in full with low risk of bias; / indicates partial reporting with unclear risk of bias; - indicates no reporting with high risk of bias.
